# Continuing Bonds after Loss by Suicide: A Systematic Review

**DOI:** 10.3390/ijerph19052963

**Published:** 2022-03-03

**Authors:** Rebecca Goodall, Karolina Krysinska, Karl Andriessen

**Affiliations:** 1 Melbourne Medical School, The University of Melbourne, Parkville, VIC 3010, Australia; rngoodall@student.unimelb.edu.au; 2 Centre for Mental Health, Melbourne School of Population and Global Health, The University of Melbourne, Parkville, VIC 3010, Australia; karolina.krysinska@unimelb.edu.au

**Keywords:** continuing bonds, grief, bereavement, suicide, systematic review

## Abstract

The concept of continuing bonds as an alternative to detachment from the deceased person has gained traction in grief literature over the years. Those bereaved by suicide are likely to experience various grief reactions and may be at-risk for adverse grief and mental health outcomes. However, it remains unclear how those bereaved by suicide experience continuing bonds. To address this gap, we conducted a systematic review according to PRISMA guidelines. Searches of peer-reviewed literature in Medline, PsycINFO, Embase, Emcare, EBM Reviews, and Scopus identified 15 studies (2 quantitative and 13 qualitative) reporting on 12 samples, published between 2010 and 2021. The study quality of the quantitative studies was poor, but it was fair amongst the qualitative studies. People bereaved by suicide experienced continuing bonds across a variety of domains and reported mostly positive experiences. Factors that tended to have an impact on the expression of continuing bonds included time since bereavement, relationship to the deceased, social expectations, sex of the bereaved, and the ability of the bereaved to make meaning of the death. The review concludes that most participants reported positive experiences with continuing bonds. However, discrepancies between males and females and between those bereaved by suicide and those bereaved by other causes warrants further investigation. In addition, longitudinal community-based research involving representative samples is needed to understand the evolution and experience of continuing bonds over time in those bereaved by suicide and to inform future efforts in supporting them.

## 1. Introduction

Suicide claims the lives of over 700,000 people per year globally [1]. A recent meta-analysis of population-based studies indicated that approximately one in twenty people experience a loss by suicide in one given year and one in five do so during their lifetime, although the impact of the death may depend on the closeness of the relationship [2]. Due to the complex nature of grief after suicide, individuals bereaved by suicide have a higher risk of adverse mental health outcomes such as posttraumatic stress disorder, depression, suicidal ideation, and attempted suicide than those bereaved by other causes [3]. People bereaved by suicide also have particular needs for professional help in dealing with their grief (for example, regarding feelings of guilt and struggles with ‘why’ questions) compared to those bereaved by natural causes [4].

Initially, grief literature suggested that bereaved individuals progress through different stages of grief in order to achieve detachment from the deceased person [5]. However, Klass and colleagues [6] challenged this theory and proposed the concept of continuing bonds as an alternative to detachment. They defined continuing bonds as the presence of an ongoing inner relationship with the deceased person [6]. Still, the concept of continuing bonds has been described in cultural literature and various religions over the centuries [7]. Klass and colleagues drew attention to specific rituals which demonstrated the practice of continuing bonds long before the theory gained traction in grief literature [6,7]. They gave the example of Japanese Buddhism, in which the deceased become part of the spirit family and are accessible to the bereaved, and in Judaism, in which the deceased are remembered through prayer and the physical lighting of a candle [6,7].

Recent studies have aimed to characterise continuing bonds as positive or negative and to specify the ways in which they manifest [7]. The process of meaning-making emerged as an important tool in the formation of positive bonds [8]. Meaning-making refers to the way in which bereaved individuals process the death in a way that holds meaning for them [9]. The concept of continuing bonds has gained traction over the years, as witnessed through the development of designated instruments [10] and studies in various bereaved populations (such as after the death of a parent or a child) [7]. However, it remains unclear how continuing bonds manifest themselves in those bereaved by suicide and how this population experiences continuing bonds. To-date, no review on this topic has been conducted. Still, a better understanding of this aspect of grief in people bereaved by suicide may provide useful information for future efforts in supporting them.

This review will address this gap by synthesising and analysing the research on continuing bonds in individuals bereaved by suicide. It aims to characterise aspects of the continuing bond including how they manifest, and whether those bereaved experience them as positive or negative.

## 2. Materials and Methods

### 2.1. Search Strategy

The review adhered to PRISMA guidelines [11], and the protocol was registered in the PROSPERO database (CRD42021271971). The review involved systematic searches of the following databases: Medline, PsycINFO, Embase, Emcare and EBM Reviews (all accessed via Ovid), and Scopus. The search in Medline comprised MeSH and text words: (Continu* bond*.mp OR continu* relationship*.mp OR continu* connection*.mp OR Ongoing bond*.mp OR Ongoing relationship*.mp OR Ongoing connection*.mp OR meaning making.mp OR Memorial*.mp OR Ritual*.mp) AND (Grie*.mp OR Grief/ OR Mourn*.mp OR Bereav*.mp OR Bereavement/ OR Suicide bereave*.mp OR Bereave* by suicide.mp OR loss by suicide.mp OR suicide loss survivor*.mp) AND (suicide.mp OR Suicide/). A similar search string including headings and keywords was used in the other databases.

**Figure 1:**
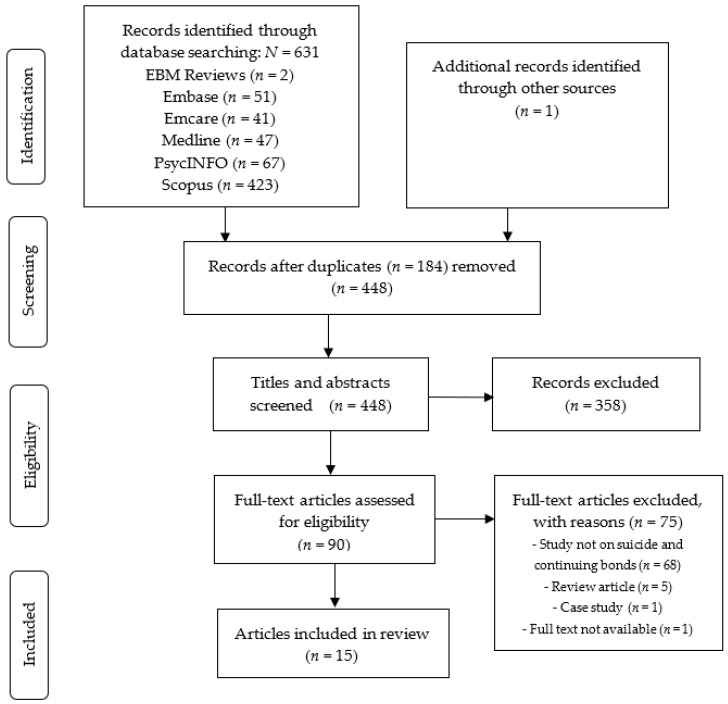
PRISMA Flow Diagram.

One researcher (R.G.) conducted the search in August 2021. It was limited to publications in English but not by date of publication. Two researchers (R.G., K.A.) assessed titles and abstracts for eligibility and any discordance was resolved through discussion with the third researcher (K.K.). The same two researchers then assessed the full text of potentially relevant studies using the inclusion/exclusion criteria. Researcher R.G. hand-searched the references of the included studies and conducted a forward-citation search in Google Scholar to identify any further studies. Figure 1 summarises the search strategy.

### 2.2. Inclusion and Exclusion Criteria

Studies were included if: (1) the study population consisted of people bereaved by suicide; (2) the study provided empirical qualitative and quantitative data on continuing bonds with an individual lost to suicide; (3) the study was published in the English language; and (4) the study was published as a paper in a peer-reviewed journal.

The review excluded: (1) studies not providing data specifically on people bereaved by suicide; (2) studies not providing data on continuing bonds in the context of suicide bereavement; (3) studies based on other methods, such as case studies, reviews and opinion paper; (4) studies not written in English; and (5) studies which were not peer-reviewed. Unclarity on the inclusion of three studies was resolved through discussion with the third researcher (K.K.).

### 2.3. Data Extraction

Two researchers (R.G., K.A.) independently extracted the following data: author, year and location of study, eligibility criteria, sample size, participants’ sex, age, time since bereavement and relationship to the deceased, setting, study design, and main findings. 

### 2.4. Quality Assessment

Two researchers (R.G., K.A.) independently conducted the quality assessment and resolved disagreements through discussion. No eligible study was excluded based on its quality. Quantitative studies were assessed using the Newcastle-Ottawa Quality Assessment Form for Cohort Studies [12], comprising eight items across three domains: (1) selection (four items), (2) comparability (one item), and (3) outcome (three items). Scores in each domain were totalled to determine study quality as good, fair, or poor. The interrater reliability was high (κ = 0.82). 

The qualitative studies were assessed using the Consolidated Criteria for Reporting Qualitative Research (COREQ) [13] consisting of thirty-two items across three domains: (1) research team and reflexivity (eight items), (2) study design (fifteen items), and (3) analysis and findings (nine items). For each study, the number and percentage of items satisfied within each domain and across all domains was calculated. The interrater agreement was high (κ = 0.92).

## 3. Results

### 3.1. Study Characteristics

The searches identified fifteen studies reporting on twelve samples. Five studies were conducted in Australia [14,15,16,17,18], three in the USA [9,19,20], three in the UK [21,22,23], two in Israel [24,25], one in Canada [26], and one in Switzerland [27]. There were two quantitative and thirteen qualitative studies. The quantitative studies collected data via online questionnaires [19,24]. One also utilised the Two-Track Bereavement Questionnaire (TTBQ) [10] to measure the level of functioning of bereaved individuals and their relationship with the deceased person [24]. Eleven of the thirteen qualitative studies involved semi-structured or in-depth interviews either in-person or via telephone, one utilised a survey [20] and one collected data through a series of workshops over twelve weeks [18].

The quantitative studies had sample sizes ranging from *N* = 159 [24] to *N* = 1301 [19], whilst qualitative studies had sample sizes ranging from *N* = 7 [14] to *N* = 50 [27], with the exception of Jahn [19,20] who used the same data set for both a quantitative and qualitative study. Table 1 and Table 2 summarise the quantitative and qualitative studies, respectively.

A large percentage (67–89%) of participants in most studies (*n* = 9) were female [16,19,20,21,22,24,25,26,27]. Three studies had equal numbers of both sexes [15,17,23], two had slightly more male (57–60%) than female participants [9,14], and one study did not report the sex of participants [18]. Across studies, the age range was between 8 and 85 years [19,23].

Whilst most studies included a broad range of relationships between the bereaved individuals and the deceased, six focused on a specific relationship, including bereaved parents (*n* = 3) [15,16,17], siblings (*n* = 2) [14,25], and children (*n* = 1) [23].

Time since death ranged between two months and over forty-five years [18,24]. Two studies focused on the grief reactions from five months onwards [21,22], whilst others looked at continuing bonds and grief reactions on a longer-term from five years onward [9] or within a ten-year period [14]. Eight studies did not limit participants by time since loss [16,19,20,23,24,25,26,27], and three included longitudinal data [15,17,18].

### 3.2. Quality Assessment

Appendix A presents the methodological quality of the quantitative studies, and the two included studies received a rating of ‘poor’ quality. One study [24] scored well in the ‘comparability’ domain, but both studies [19,24] tended to score poorly in the ‘selection’ and ‘outcome’ domains by using selected samples and relying on self-reported outcomes. Appendix B outlines the quality assessment of the 13 qualitative studies. The studies satisfied between 34% [22] and 75% [15,17] of the COREQ criteria [13]. Most studies reported only few items across the ‘research team and reflexivity’ domain (on average 35% of items were reported). However, most items (on average 63%) in both the ‘study design’ and ‘analysis and findings’ domains were reported.

### 3.3. Study Findings

#### 3.3.1. In What Way Can Continuing Bonds Manifest?

Findings from the included studies showed that continuing bonds manifested or were experienced in various ways. Quantitative data showed that 63% of participants reported having spiritual experiences [19]. The most common experiences included dreams about the deceased (73.4%), feeling the presence of the deceased (53%), and profound coincidences (40.3%) [19]. In qualitative studies, participants reported the use of rituals and memorials (both physical and online) [9,16,21,23], spiritual and religious beliefs [9,26,27], metaphysical experiences [20,26], physical objects [9], and suicide-related thoughts and behaviours, such as a desire to join the deceased and/or understand what they went through [9].

Suicide bereaved individuals tended to experience continuing bonds across multiple domains both concurrently and over time [15,25]. It was common to experience continuing bonds across a continuum, for example, engaging in both public and private expressions of continuing bonds [25]. A public manifestation may include participation in ceremonies or rituals such as a funeral whilst a private manifestation may include writing to or talking to the deceased [25]. Although the process of developing continuing bonds was never described as linear, time since bereavement did appear to affect how the bonds were experienced. Entilli and colleagues [15] and Ross and colleagues [17] described experiences with continuing bonds as they appeared at 6, 12, and 24 months, observing that memorialisation and intrusive thoughts were present early on whereas paranormal beliefs and the maintenance of the relationship with the deceased were new themes at 24 months. Similarly, Sands and colleagues [18] reported themes as they appeared during workshops spanning over 12 weeks. Participants experienced thoughts around ‘why’ the person had died by suicide before reconstructing the death story and repositioning the relationship in order to establish positive ongoing bonds [18]. Suicidal ideation with a desire to join the deceased and/or to understand what they went through was another common initial reaction that typically subsided over time [9,14].

Qualitative studies found that most participants wanted to experience continuing bonds and actively pursued them [14,26]. They achieved this through the creation of online memorials [21,22], visiting places that were frequented by the deceased [16], keeping objects that belonged to the deceased [26], actively reminiscing about the deceased [26], and writing to the deceased [15]. However, some participants found that the social expectations of grief and the stigma associated with suicide limited their public expression of continuing bonds, forcing them to express their grief privately [16,21,22].

There were differences in the types of bonds experienced according to sex and type of relationship to the deceased person [15,20,21]. A qualitative study found that female participants and those who had a close pre-death relationship with the deceased family member or partner had more spiritual experiences with the deceased [20]. Females also created more online memorials than males [21]. Conversely, males appeared to engage more in avoidant coping strategies and would express their grief less openly [15]. Though children may experience continuing bonds in similar domains as adults, research suggests they tend to interpret their experiences differently and this varies depending on age [23].

#### 3.3.2. Continuing Bonds as a Positive or Negative Experience

Participants generally perceived continuing bonds as positive experiences [16,17,19,20,21,27], which they associated with comfort and hope [26]. Although less common, negative experiences were those that were beyond the control of the bereaved individuals and included subconscious thoughts or feelings [25], spiritual or metaphysical experiences [26], or the unexpected discovery of facts relating to the deceased that were not in-line with the perceptions of the bereaved [21]. A quantitative study [19] demonstrated that 74.5% of participants interpreted spiritual experiences with the deceased as helpful whilst only 4.8% viewed them as harmful [19]. However, another quantitative study comparing outcomes of those bereaved by suicide with those bereaved by sudden or expected death found that those bereaved by suicide had lower levels of close positive relationships with the deceased both pre- and post-death, including less expression of continuing bonds [24].

## 4. Discussion

This review aimed to synthesise the research literature concerning continuing bonds in people bereaved by suicide, including how they manifest and whether the bereaved experienced them as positive or negative. The review identified 15 studies reporting on 12 samples. Factors that affected the way in which continuing bonds manifested included time since death, type of relationship, societal expectations, sex of the bereaved person, and their ability to make meaning of the death. In all but one study [22], participants reported mostly positive thoughts, feelings and experiences regarding continuing bonds.

### 4.1. Factors That Affect the Manifestation of Continuing Bonds

Aside from two longitudinal studies [15,17], there appears to be little research on the effect of time since bereavement on the manifestation of continuing bonds in those bereaved by suicide. Participants from one study [9] reported that the intensity of the bonds had not lessened up to 10 years following the suicide. This is supported by a quantitative study [24] which found that when compared to those bereaved by sudden or expected deaths, those bereaved by suicide tended to maintain more intense bonds for a longer period of time and consequently took longer to engage in ‘meaning-making’. These findings are important due to the strong association between intense grief and less positive personal transformation [24], highlighting the need for more longitudinal, comparative data.

Societal beliefs and expectations may impact on the manifestation of continuing bonds in several ways [16,21,22,27]. Some participants felt they were forced to express their ongoing relationship more privately due to the stigma surrounding suicide [9,21], and the expectation to resolve grief [16]. Our review revealed that suicidal ideation [9,14] in suicide bereaved individuals can be a way to reconnect with the deceased or to understand what they went through, suggesting that suicidal ideation may be a manifestation of continuing bonds in those bereaved by suicide. Interestingly, participants claimed that this process aided their grief work by helping them to make sense of the suicide; however, reported they felt discouraged from sharing these thoughts with others [9]. These findings illustrate the importance of addressing suicidal thoughts in a constructive manner as opposed to inadvertently reinforcing suicide stigma by silencing them [9].

The stigma associated with suicide in the context of religions may be particularly strong, with some bereaved individuals choosing to hold private ceremonies following a suicide death [27,28]. This is supported by a qualitative systematic review that found that religious individuals often isolate themselves due to feelings of shame and suicide-related stigmatisation emanating from religious doctrines [29]. Interestingly, taking part in religious ceremonies was common practice even in those participants who identified as agnostic [27]. A possible explanation for this may be that societal and cultural expectations dictate how we grieve, insofar as rituals such as funerals are expected in western culture [30]. Future studies conducted in different cultures may shed light on the expression of continuing bonds with regards to spiritual and religious themes and the possible stigma in this context.

Referring to the ‘Dual Process Model of Coping with Bereavement’ [31], studies reported differences between males and females with females engaging in more ‘loss-oriented’ behaviours, including continuing bonds, and males engaging in more ‘restoration-oriented’ behaviours, including learning new skills [15]. Whilst it may be the case that males experience continuing bonds to a lesser extent than females and/or in different domains [32], literature suggests that societal perceptions of gender and masculinity may influence the expression of grief, and grief after suicide, in males [33], resulting in males engaging in restoration-oriented activities as a distraction from or expression of their grief [33]. This view is supported by Entilli and colleagues [15] who found that fathers avoided discussing their feelings regarding the loss by suicide. Future studies may further compare the experience of continuing bonds between males and females and inform services to be directed accordingly.

The process of ‘meaning-making’ was mentioned throughout the reviewed studies. It refers to the ability of the bereaved person to make sense of or find meaning in the death [8,34] and has been widely recognised in grief literature [8,34]. Milman and colleagues described meaning-making as a method of alleviating the cognitive dissonance caused by the death through purposeful reflection [34]. They highlight the importance of differentiating this process from rumination as the latter fails to negotiate this discrepancy and is characterised by passive, repetitive and negative thoughts [34]. Our review indicated that those who were able to make meaning of the suicide experienced more positive continuing bonds whilst those who were unable to make meaning experienced more negative continuing bonds. In addition, high resilience characteristics and a strong social support network tended to predict less intense continuing bonds and higher levels of posttraumatic growth [24].

### 4.2. Continuing Bonds as a Positive or a Negative Experience

Most participants in the included studies reported positive feelings experienced with continuing bonds [16,18,23], a finding that is mirrored by those of general bereavement studies [35,36]. However, a quantitative study comparing different forms of bereavement indicated that those bereaved by suicide reporting lower levels of close and positive relationships with the deceased both pre-and post-death [24]. The incorporation of multiple factors, including close and positive, and pre-and post-death relationships, make it hard to accurately compare these results with other studies. As demonstrated by Leichtentritt and colleagues [25], a ‘close’ relationship is not necessarily synonymous with a ‘positive’ one. Likewise, as suggested throughout this review, the process of meaning-making transforms the relationship so that the pre- and post-death relationships may not be equivalent. Further studies comparing experiences of continuing bonds between those bereaved by different causes may further clarify how to understand these experiences.

Negative experiences occurred when continuing bonds in the form of memories or objects faded or were lost [14,21], when participants were not able to make meaning of the loss [15], or when aspects of continuing bonds were beyond the control of the bereaved [22,25]. Participants experienced feelings of distress at fading memories of the deceased [14], or due to the sudden and unexpected disappearance of online memorials [21], which was described as a ‘double loss’ [22]. Some expressed apprehension and guilt as contributing to the continuing bonds as they did not want to abandon the deceased person [10]. Additionally, participants experienced cognitive dissonance when their religious beliefs were in opposition to the actions of the deceased [27], or upon the discovery of new information about the deceased that was contradictory to their own internal representation of the deceased person [21,22]. These psychological discrepancies were found to hinder the ability of the bereaved person to make meaning of the death and enhanced negative feelings associated with continuing bonds [9]. Further research may clarify in which circumstances it may be more beneficial for the bereaved person to relinquish than to maintain the bond [35,36].

Participants utilised several tools and behaviours to create or maintain positive continuing bonds. Many studies observed the tendency of participants to omit certain aspects of the deceased personality or behaviour when recalling memories of them [23], or to hold more symbolic or fictitious representations of the deceased [25,27]. Some bereaved individuals favoured online memorials due to them being more interactive than traditional mourning objects [21]. This is supported by literature reporting that this allows the bereaved to explore the relationship in more depth and to discover new things about the deceased, thus enabling the relationship to evolve [37]. Nonetheless, a potential negative aspect of online grief-related activities is the ease with which those bereaved can become preoccupied with the relationship, hindering the meaning-making process [21,38].

Positive and negative experiences with continuing bonds were often intertwined. Wood [23] found that negative thoughts about the deceased’s personality or behaviour initially evoked distress but with reflection and perspective, understanding and personal growth could be achieved. This was echoed by Maple [16], who found that parents could initially be distressed at the ongoing ‘presence’ of their deceased child but in time came to enjoy these experiences. Nonetheless, participants reported a bitter ‘aftertaste’ associated with positive thoughts about the deceased as they tended to trigger difficult emotions and memories [23]. Remembering the more holistic picture (both good and bad) was beneficial in making sense of the suicide and relieving responsibility and guilt in those bereaved by suicide [23,39].

### 4.3. Limitations

The studies included in the review and the review itself entailed a few limitations. Most studies were qualitative, or cross-sectional (of relatively poor quality) with primarily female participants from western countries, making the impact of culture, sex and time since bereavement difficult to discern. This highlights the need for international research involving representative samples. Also, further studies involving control groups or adopting community-based longitudinal designs may enable researchers to capture the experiences of continuing bonds over time. While the review involved searches in six databases, future reviews can broaden the scope by including more databases as well as grey literature, which may increase, for example, the likelihood of finding negative experiences in the context of continuing bonds.

### 4.4. Implications

A better understanding of continuing bonds in suicide bereavement may inform future interventions and enable service providers to deliver more accurate and targeted support. In particular, the review highlights the substantial impact that societal expectations and stigma continue to have on the experience of grief in suicide bereaved individuals [40]. The findings of this review may direct future studies and help to characterise the experiences of continuing bonds in people bereaved by suicide with greater clarity.

## 5. Conclusions

People bereaved by suicide commonly experience continuing bonds and generally interpret these as positive experiences. Factors such as time since bereavement, social and cultural expectations, sex of the bereaved person, and the ability of the bereaved to make meaning of the death may influence how continuing bonds manifest and whether they are experienced as positive or negative (although further research is needed). Postvention efforts should consider the process of meaning-making in creating the basis for positive continuing bonds and should attempt to address the stigma and societal expectations surrounding suicide bereavement. Future studies should involve representative samples, compare with continuing bonds after other causes of death, and investigate continuing bonds in suicide bereavement longitudinally.

## Figures and Tables

**Table 1:** Summary of quantitative studies.

Author, Year, Location	Eligibility Criteria	Sample Size	Male/Female	Age	Time since Bereavement	Relationship to Deceased	Setting	Study Design	Outcome Measures	Main Results
Levi-Belz (2017) Israel [24]	>18 y/o, identify as a bereaved family member. Excl.: <15 y/o at time of death, inability to speak or write in Hebrew or English.	*N* = 159 suicide loss survivors = 58, sudden-death bereavers = 48, expected-death bereavers = 53 (F = 81%)	M = 30 F = 129 suicide loss survivors: M = 13, F = 45 sudden-death bereavers: M = 6, F = 42 expected-death bereavers: M = 11, F = 42	18–73 suicide loss survivor M = 40.5, SD = 15.6, sudden-death bereavers M = 35.7, SD = 13.1, expected-death bereavers M = 33.1, SD = 12.3	2 m–45 y (M = 95 m, SD = 105.46 m)	25 parents, 11 children, 26 siblings, 30 spouses, and 44 close family members, 9 unknown	Online questionnaire	Cross sectional	Level of functioning: Two-track bereavement questionnaire (TTBQ), Post-traumatic grief level: Stress-Related Growth Scale (SRGS)	Suicide-loss survivors reported lower levels of close positive continuing bonds with the deceased than did participants from the sudden- and expected-death bereaved groups. Intense grief and preoccupation were associated with less positive personal transformation, particularly among the suicide bereaved. This suggests that continuing bonds in those bereaved by suicide are more likely to be experienced negatively than in those bereaved by other causes.
Jahn et al. (2014) USA [19]	Survivors of suicide loss	*N* = 1301	M = 135 F = 1155 U = 11	40–85	1 y–3 y (28.9%; *n* = 376), <1 y (25.7%; *n* = 334), 4 y–10 y (22.0%; *n* = 286), 11 y–20 y (12.2%; *n* = 159), and >20 y (8.2%; *n* = 107).	Parent (*n* = 362), sibling (*n* = 235), spouse/partner (*n* = 204), child (*n* = 180), friend (*n* = 112), niece/nephew (*n* = 21), grandchild (*n* = 9), another relationship (*n* = 119).	Two questionnaires—one demographics and one about experience of suicide. Both containing both qualitative and quantitative info	Descriptive statistics used to explore characteristics of loss and chi squared to examine demographical differences between responses	Two questionnaires: (i) demographic questionnaire, (ii) spiritual experiences of survivors of suicide, including questions about type and frequency of spiritual experiences	Most (*n* = 819, 63%) of those bereaved by suicide reported after-death spiritual experiences including dreams, feeling the presence of the person and profound coincidences. These experiences are often regarded as positive with 74.5% of participants (*n* = 610) finding them ‘helpful’ and only 4.8% (*n* = 39) viewing them as ‘harmful’ (19.9%, *n* = 163 saw them as neither helpful nor harmful). Being female and having a closer pre death relationship (family member as opposed to friend or client) was positively associated with spiritual experiences.

**Table 2:** Summary of qualitative studies.

Author, Year, Location	Eligibility Criteria	Sample Size	Male/Female	Age (Years)	Time Since Bereavement	Relationship to Deceased	Setting	Study Design	Main Results
Adams et al. (2019) AUS [14]	Bereaved by suicide of sibling (sibling <20 y/o) in past 10 yrs	*N* = 7	M = 4 F = 3	20–27 at time of interview (16–23 at time of suicide)	Average time = 3 y 9 m	Sibling	Telephone interview	Interpretive phenomen-ological analysis (IPA)	Identified 4 main themes: (a) the process of grief, (b) grief interactions (within families and outside), (c) continuing bonds, and (d) meaning-making and growth through grief.
Bailey et al. (2015) UK [21]	Family members and friends who own suicide memorial sites	*N* = 11	M = 3 F = 8	20–60	5 m–4 y	Parent, siblings or friends	Face-to-face interviews—semi-structured narrative style	Qualitative interpretative approach, combining constant comparison techniques with thematic analysis	The most common motivating factor for starting a memorial page was to ‘keep the deceased alive’ and maintain a connection. Participants found that they were able to better construct and refine relationships with the deceased using online memorials. Whilst most people had positive experiences with memorial sites, the dangers of becoming overly attached and experiencing compounding grief or ‘double loss’ was highlighted.
Bell et al. (2015) UK [22]	Individuals who had set up or were managing memorial sites for those who dies by suicide	*N* = 11	M = 3 F = 8	20–60	5 m–4 y	Parent, siblings or friends	Individual interviews	Qualitative interpretative approach, combining constant comparison techniques with thematic analysis	Provided insight into how online memorialisation allows more flexibility and depth in the exploration of grief than can traditional mourning objects—this allows users to reminisce on positive aspects of deceased life but can elicit negative experiences as users can’t control how memories are framed.
Castelli Dransart (2018) Switzerland [27]	Suicide-survivors: A person was considered as a survivor of suicide if: (1) he/she self-qualified as such; (2) he/she felt emotionally close to the deceased; and (3) his/her life had been disrupted by a suicide (self-perception). >18 y/o, able to speak Italian, French or German	*N* = 50	M = 11 F = 39	14–73	<12 m–16 y	18 mothers, 5 fathers, 10 sisters, 3 brothers, 3 daughters, 1 son, 7 partners, 1 aunt, 2 friends	Face-to-face in depth interviews conducted by author or mental health carer, either at home or location chosen by bereaved	Grounded Theory using constant comparison of data and 3 steps of coding: open, axial & selective	Suicide triggered spiritual and religious thoughts and experiences for most participants. Even those who claimed to be atheist or agnostic noted religious rituals and spiritual symbols as being important contributors in forging and maintaining a continuing bond with the deceased and in honouring their memory. Interviewees believed loved ones continued to exist in an alternative dimension or space (regardless of religion).
Entilli et al. (2021) AUS [15]	Parents who had lost a child by suicide less than 6 months prior to starting the study	*N* = 14 at 6 and 12 months. *N* = 11 at 24 months	M = 7 F = 7 (6 and 12 months). M = 6 F = 5 (at 24 months)	Female mean = 60.1 years, range = 50–78 years and male mean = 59.9 years, range = 50–68 years	6 m, 12 m and 24 m	Parents who had lost a child (aged 15–51) by suicide. Ten were bereaved of sons and four bereaved of daughters	Semi-structured interviews either phone or face-to-face	Longitudinal study using thematic analysis	Three key themes were identified in an earlier analysis (at 6 and 12 months post loss): searching for answers and sense-making, coping strategies and support, and finding meaning and purpose. Further exploration of these themes at 24 months revealed significant differences between mothers and fathers with the latter adopting more maladaptive coping strategies. Maintaining the relationship with the deceased and paranormal experiences were new themes at 24 months (not present at 6 and 12 months) and a shift from brooding to reflection/sense-making was seen at 24 months. The adaptation process was fluctuating and dynamic.
Gall et al. (2015) Canada [26]	Individuals who had personal experiences of suicide bereavement	*N* = 15 (11 bereaved and 4 mental health workers)	M = 2 F = 9 (bereaved) M = 1 F = 3 (MHW)	Mean age was 49 (bereaved) and 53 (MHW)	Mean = 13 y, minimum of 2 y	The deceased persons were: four sons, two fathers, two close friends, two uncles and one mother.	Semi-structured interviews	Phenomenological approach, thematic analysis	Individuals had difficulty reconciling the suicide death of a loved one with their religious views. This often led to a personally defined spirituality rather than a complete loss of faith. Many individuals (also non-religious) found meaning in the belief of an afterlife and/or felt hopeful that the deceased was in a better place and would someday reconnect with them. Engagement in activities to maintain a bond were common.
Hunt et al. (2019) USA [9]	>18 y/o, self-identified as suicide loss survivor, >5 years since death	*N* = 10	M = 6 F = 4	30–72 (mean = 47.6)	5 y–30 y (mean = 18.6 y)	5 siblings, 2 partners, 2 parents and one participant who had lost a sibling, grandfather and cousin	Face-to-face semi-structured interviews at participants homes	Thematic analysis informed by grounded theory	Three major themes were identified: - one harmful (feelings of responsibility) - one helpful (making meaning) to the process of suicide bereavement. - one that helped shift from harmful to helpful (social support) Participants did not identify with the Kubler-Ross stages of grief theory.
Jahn et al. (2018) USA [20]	Any person who identified as bereaved by suicide	*N* = 1301	M = 135 F = 1155 U = 11	40–85	1 y–3 y (28.9%; *n* = 376), <1 y (25.7%; *n* = 334), 4 y–10 y (22.0%; *n* = 286), 11 y–20 y (12.2%; *n* = 159), and >20 y (8.2%; *n* = 107).	Anyone bereaved by suicide	Two questionnaires—one demographics and one about spiritual experiences after suicide bereavement. Both containing both qualitative and quantitative info	Inductive thematic analysis	Nine main themes were identified: (1) a helpful sense of comfort; (2) a helpful sense of connection with the deceased; (3) intense sadness evoked by the spiritual experiences; (4) confusion regarding the spiritual experiences; (5) negative reminders of the deceased or negative meanings of spiritual experiences; (6) evidence of an afterlife; (7) general importance of the spiritual experiences’ meaning; (8) impact of and on religious beliefs; and (9) others’ responses to disclosure of suicide or spiritual experiences. Generally, participants found spiritual experiences aided in healing and transformation and were regarded as positive.
Leichtentritt et al. (2015) Israel [25]	Having experienced the loss of a sibling to suicide and the death having occurred at least five years prior to the interview	*N* = 9	M = 3 F = 6	29–63	5 y–37 y	Sibling	In-depth interviews	Relational dialect theory and narrative analysis used.	Five characteristics of the post death relationship were identified, each existing along a continuum: (1) concrete-symbolic (2) dynamic-static (3) conscious-unconscious (4) personal-public (5) monologue-dialogue Findings suggest that labelling post death relationships as ‘adaptive’ or ‘maladaptive’ is simplistic. Bereavement can be better understood when plotted within the 5 continua.
Maple et al. (2013) AUS [16]	Parents who lost a child by suicide	*N* = 22	M = 6 F = 16	NA	6 m–>26 y	22 parents (6 fathers and 16 mothers) from 18 families bereaved of 15 sons and 3 daughters. 14 participated individually and 4 as couples	In-depth interviews, mostly face-to-face (one phone)	Narrative Inquiry, recursive technique used to explore in more depth.	Contrary to traditional grief literature, it was found that parents needed to maintain a relationship with their deceased child. Manifestations of continuing bonds varied between parents. Commencing with the funeral, parents began developing rituals ensuring that their child’s life, and not the manner of death, was celebrated. Some participants were limited in their expression of grief due to social pressure to resolve grief.
Ross et al. (2018) AUS [17]	Parents bereaved by suicide loss of their child 6 months prior to commencement of study	*N* = 14	M = 7 F = 7	50–78 (female mean = 60.1 years and range = 50–78, male mean = 59.9 years and range = 50–68)	6 m and 12 m	Parents who had lost a child (aged 15–51) by suicide. Ten were bereaved of sons and four bereaved of daughters	Individual, semi-structured interviews either face-to-face or telephone	Longitudinal study with inductive qualitative approach.	Identified three key themes (searching for answers and sense-making, coping strategies and support, and finding meaning and purpose) in parental responses to suicide bereavement. The phases of sense-making and meaning-making experienced by participants and the range of both adaptive and maladaptive coping strategies indicated that adapting to bereavement is a dynamic and fluctuating process.
Sands et al. (2010) AUS [18]	Adults >19 y/o, bereaved through the suicide death of a significant person in their lives	*N* = 16	NA	Aged 19+	>2 m	Family members or spouses: partner grieving a partner, parent grieving a child, sibling grieving a sibling, and adult child grieving a parent	A series of workshops delivered over 12 weeks and 30 h–involved discussion, artwork, grief rituals and journal writing	Generic thematic analysis	Identified 3 core themes that assisted in meaning making in relationships with the themselves, the deceased, and with others. The 3 themes were: (i) Intentionality (‘tying on the shoes’ or adopting the perspective of the deceased), (ii) Reconstruction (‘walking in the shoes’), (iii) Repositioning (‘taking off the shoes’)
Wood et al. (2012) UK [23]	8–15 y/o (M = 11.80, SD = 2.57), whose parent had died by suicide within the previous 13 to 53 months	*N* = 10	M = 5 F = 5	8–15	13 m–53 m (M = 33.4, SD = 17.44)	Eight children bereaved of a father and two bereaved of a mother	Semi-structured interviews, face-to-face at participants’ homes	Interpretative Phenomen-ological Analysis	Found 3 main themes: thinking about the deceased; coping strategies; and connecting to the deceased. Highlighted differences in childhood suicide bereavement as well as factors which may influence adaptiveness.

## Appendix A

**Table A1:** Quality assessment ^1^ of quantitative studies.

Topic	Jahn & Spencer-Thomas, 2014 [19]	Levi-Belz, 2017 [24]
**Selection**		
(1) Representativeness of the exposed cohort		
(a) Truly representative (one star)		
(b) Somewhat representative (one star)		
(c) Selected group	X	X
(d) No description		
(2) Selection of the non-exposed cohort		
(a) Drawn from the same community as the exposed cohort (one star)	N/a	X
(b) Drawn from a different source		
(c) No description		
(3) Ascertainment of exposure		
(a) Secure record (e.g., surgical record) (one star)		
(b) Structured interview (one star)		
(c) Written self-report	X	X
(d) No description		
(e) Other		
(4) Demonstration that outcome of interest was not present at start of study		
(a) Yes (one star)	X	X
(b) No		
**Comparability**		
(1) Comparability of cohorts on the basis of the design or analysis controlled for confounders		
(a) The study controls for age, sex and marital status (one star)		X
(b) Study controls for other factors (list) (one star)		X
(c) Controls are not comparable		
**Outcome**		
(1) Assessment of outcome		
(a) Independent blind assessment (one star)		
(b) Record linkage (one star)		
(c) Self-report	X	X
(d) No description		
(e) Other		
(2) Was follow-up long enough for outcomes to occur		
(a) Yes (one star)	X	X
(b) No		
Indicate the mean duration of follow-up and a brief rationale for the assessment above	Range <1–20+ years	Range 2–540 months
(3) Adequacy of follow-up of cohorts		
(a) Complete follow-up, all subjects accounted for (one star)		
(b) Subjects lost to follow-up unlikely to introduce bias, number lost less than or equal to 20% or description of those lost suggested no different from those followed (one star)		
(c) Follow-up rate less than 80% and no description of those lost		
(d) No statement	X	X
**Stars**		
Selection	1	2
Comparability	0	2
Outcome	1	1
**Rating**	Poor	Poor

^1^ Newcastle-Ottawa Quality Assessment Form for Cohort Studies [12]. Note: A study can be given a maximum of one star for each numbered item within the Selection and Outcome categories. A maximum of two stars can be given for Comparability. Thresholds for converting the Newcastle-Ottawa scales to AHRQ standards (good, fair, and poor): Good quality: 3 or 4 stars in selection domain AND 1 or 2 stars in comparability domain AND 2 or 3 stars in outcome/exposure domain. Fair quality: 2 stars in selection domain AND 1 or 2 stars in comparability domain AND 2 or 3 stars in outcome/exposure domain. Poor quality: 0 or 1 star in selection domain OR 0 stars in comparability domain OR 0 or 1 stars in outcome/exposure domain.

## Appendix B

**Table A2:** Quality assessment ^1^ of qualitative studies.

	Topic	Adams et al., 2019 [14]	Bailey et al., 2015 [21]	Bell et al., 2015 [22]	Castelli, 2018 [27]	Entilli et al., 2021 [15]	Gall et al., 2015 [26]	Hunt et al., 2019 [9]	Jahn et al., 2018 [20]	Leichtentritt et al., 2015 [25]	Maple et al., 2013 [16]	Ross et al., 2018 [17]	Sands et al., 2010 [18]	Wood et al., 2012 [23]
	**Domain 1: Research team and reflexivity**													
	*Personal characteristics*													
1	Interviewer/facilitator	p. ^2^ 324–325			p. 4			p. 337			p. 59	p. 3	p. 103	
2	Credentials	p. 325				p. 2						p. 2	p. 121	
3	Occupation	p. 325				p. 2				p. 1107	p. 59	p. 3	p. 121	
4	Gender	p. 324				p. 2		p. 337				p. 2	p.105/121	
5	Experience and training	p. 325			p. 4	p. 2				p. 1107	p. 59	p. 2	p. 121	
	*Relationship with participants*													
6	Relationship established				p. 4									p. 878
7	Participant knowledge of the interviewer							p. 337				p. 3		
8	Interviewer characteristics	p. 324–325						p. 337			p. 59	p. 2		
	**Domain 2: Study design**													
	*Theoretical framework*													
9	Methodological orientation and theory	p. 325	p. 75	p. 379	p. 3–4	p. 3	p. 102	p. 336	p. 6	p. 1105–1106	p. 59	p. 2–3	p. 99–102	p. 877
	*Participant selection*													
10	Sampling	p. 325	p. 74	p. 378	p. 4	p. 2–3	p. 101	p. 336	p. 5	p. 1105	p. 59	p. 2	p. 103	p. 877
11	Method of approach	p. 325	p. 74	p. 378	p. 4	p. 2	p. 101–102	p. 336–337	p. 5–6	p. 1105	p. 59	p. 2	p. 103–104	p. 877–879
12	Sample size	p. 325	p. 74	p. 378	p. 5	p. 2	p. 101	p. 336	p. 5	p. 1105	p. 59	p. 2–3	p. 103	p. 877
13	Non-participation	p. 325				p. 2–3								
	*Setting*													
14	Setting of data collection	p. 325	p. 75		p. 4	p. 2		p. 336	p. 5		p. 59	p. 2	p. 103	p. 878
15	Presence of non-participants										p. 59			
16	Description of sample	p. 325	p. 74	p. 378	p. 5	p. 2–3	p. 101	p. 336	p. 5	p. 1105	p. 59	p. 2–3	p. 103	p. 877–878
	*Data collection*													
17	Interview guide	p. 325	p. 74–75	p. 378	p. 3–4	p. 2	p. 102	p. 337	p. 5–6	p. 1106	p. 59	p. 2	p. 103–104	p. 878–879
18	Repeat interviews					p. 2	p. 102					p. 2		
19	Audio/visual recording	p. 325	p. 75	p. 378	p. 4	p. 2	p. 102	p. 337		p. 1106	p. 59	p. 2	p. 103	p. 879
20	Field notes				p. 4					p. 1107				
21	Duration	p. 325	p. 75		p. 4	p. 3	p. 101			p. 1106	p. 59	p. 2	p. 103–104	p. 878
22	Data saturation				p. 5	p. 2		p. 338				p. 2–3		
23	Transcripts returned				p. 5									
	**Domain 3: Analysis and findings**													
	*Data analysis*													
24	Number of data coders	p. 325				p. 3	p. 102	p. 337	p. 6			p. 3	p. 105	p. 879
25	Description of the coding tree	p. 326				p. 3–6		p. 337–338						
26	Derivation of themes	p. 325	p. 75	p. 378	p. 4	p. 3	p. 102	p. 337	p. 6	p. 1107	p. 59	p. 3	p. 105–106	p. 879
27	Software				p. 4	p. 3			p. 6			p. 3		
28	Participant checking				p. 5			p. 337						
	*Reporting*													
29	Quotations presented	p. 326–329	p. 76–81	p. 380–385	p. 6–14	p. 3–6	p. 102–108	p. 338–342	p. 6–10	p. 1108–1114	p. 60–64	p. 3–6	p. 107–113	p. 880–888
30	Data and findings consistent	p. 329–331	p. 81–83	p. 383–386	p. 14–17	p. 6–10	p. 108–110	p. 342–343	p. 10–11	p. 1115–1117	p. 65–67	p. 7–8	p. 114–117	p. 888–894
31	Clarity of major themes	p. 326–329	p. 78–81	p. 379–382	p. 5–14	p. 3–6	p. 102–108	p. 338–342	p. 6–10	p. 1107–1115	p. 60–64	p. 3	p. 106–114	p. 880–888
32	Clarity of minor themes					p. 3–6		p. 338–342					p. 105–106	p. 880–888
	**Rating**													
	Domain 1: Research team and reflexivity	6/8 (75%)	0/8 (0%)	0/8 (0%)	3/8 (38%)	4/8 (50%)	0/8 (0%)	4/8 (50%)	0/8 (0%)	2/8 (25%)	4/8 (50%)	7/8 (88%)	5/8 (63%)	1/8 (13%)
	Domain 2: Study design	10/15 (67%)	9/15 (60%)	7/15 (47%)	12/15 (80%)	12/15 (80%)	9/15 (60%)	9/15 (60%)	7/15 (47%)	9/15 (60%)	10/15 (67%)	11/15 (73%)	9/15 (60%)	9/15 (60%)
	Domain 3: Analysis and findings	6/9 (67%)	4/9 (44%)	4/9 (44%)	6/9 (67%)	8/9 (89%)	5/9 (56%)	8/9 (89%)	6/9 (67%)	4/9 (44%)	4/9 (44%)	6/9 (67%)	6/9 (67%)	6/9 (67%)
	Total	22/32 (69%)	13/32 (41%)	11/32 (34%)	21/32 (66%)	24/32 (75%)	14/32 (44%)	21/32 (66%)	13/32 (41%)	15/32 (67%)	18/32 (56%)	24/32 (75%)	20/32 (63%)	16/32 (50%)

^1^ Consolidated Criteria for Reporting Qualitative Research (COREQ) [13]. ^2^ In this table, “p.” refers to page numbers.
